# Dapper homolog 1 alpha suppresses metastasis ability of gastric cancer through inhibiting planar cell polarity pathway

**DOI:** 10.18632/oncotarget.13234

**Published:** 2016-11-09

**Authors:** Yuegeng Liu, Jingwan Zhang, Weifang Yu, Xiaoming Zhang, Guiqi Wang, Zengren Zhao

**Affiliations:** ^1^ Departments of Surgery, First Affiliated Hospital, Hebei Medical University, Shijiazhuang, China; ^2^ Endoscopy Center, First Affiliated Hospital, Hebei Medical University, Shijiazhuang, China

**Keywords:** DACT1α, gastric cancer, metastasis, tumor suppressor

## Abstract

Dapper homolog 1 alpha (DACT1α) is a member of DACT family and an important regulator in the planar cell polarity pathway. We aim to clarify its functional role in metastasis ability of gastric cancer. DACT1α was silenced in all gastric cancer cell lines (8/8), but expressed in normal gastric tissue. Ectopic expression of DACT1α in silenced gastric cancer cell lines (AGS, BGC823 and MGC803) by stable transfection significantly suppressed cancer cell spreading (*P* < 0.05), migration (*P* < 0.01) and invasion (*P* < 0.01). These effects were associated with downregulation of planar cell polarity pathway related genes involved in cell proliferation (PDGFB, VEGFA), adhesion (ITGA1, ITGA2, ITGA3, ITGB3) and migration/invasion (PLAU, MMP9, MCAM, Dvl-2 and JNK). DACT1α promoter methylation was detected in 205 gastric cancers and 20 normal controls by direct bisulfite genomic sequencing. DACT1α methylation was detected in 29.3% (60/205) of gastric cancer patients, but not in normal tissues. DACT1α methylation was associated with poor survival of gastric cancer patients. In conclusion, DACT1α plays a pivotal role as a potential tumor suppressor in migration and invasion of gastric cancer. DACT1α methylation may serve as a biomarker for the prognosis of gastric cancer.

## INTRODUCTION

Gastric cancer is the fourth most common cancer worldwide with 930,000 newly diagnosed cases in 2002. Gastric cancer is the third most common cause of cancer-related death in the world [1]. Since the lack of early symptoms often delays the diagnosis, 80% to 90% of patients with gastric cancer present with locally advanced or metastatic tumors that have poor survival rates [1]. Similar to other types of cancer, gastric cancer develops via a multistage process, including DNA methylation of CpG islands, post-translational modification of histones, microRNAs and nucleosome positioning [2–4]. Among them, promoter methylation is considered as one of the major mechanisms to inactivate tumor suppressor genes, thereby causing gastric carcinogenesis [5]. Promoter methylation is considered as an important hallmark of the progression and metastasis of gastric cancer. So far, many methylated genes have been identified play important roles in cell proliferation, cell cycle, apoptosis, angiogenesis, metastasis, thus contributing to the development of gastric cancer [6–10].

Dapper homolog 1 alpha (DACT1α), a member of DACT family, is an important regulator in the planar cell polarity (PCP) pathway which is an important pathway in cancer metastasis [11, 12]. It has been reported that DACT1α played an important role in regulating PCP pathway by post-translationally regulating central PCP component Dvl2 and vang-like 2 (Vangl2) and modulating PCP downstream Rac1/JNK cascade [11, 12]. Subsequent investigations have revealed that DACT1α is involved in the pathogenesis of multiple forms of malignant cancers in humans including gastric cancer [13, 14]. Hypermethylation of the DACT1 DNA promoter was frequently identified to play a regulator of tumorigenesis and prognosis in gastric cancer [13, 14]. However, the functional significance of DACT1α in regulation cell metastasis ability is still largely unclear. In this study, we mainly aimed to study the metastasis ability of DACT1α in gastric cancer.

## RESULTS

### Silence or down-regulation of DACT1α in gastric cancer cell lines

**Figure 1:**
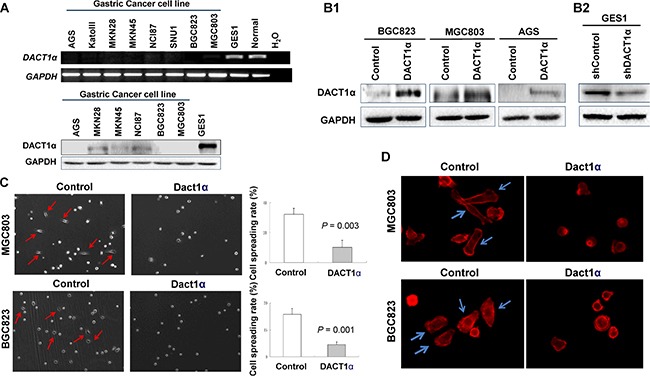
DACT1α suppressed cell spreading and F-actin formation (**A**) DACT1α expression was silenced in all gastric cancer cell lines, but expressed in normal human epithelial cell line GES1 and in normal gastric tissues. (**B1**) Ectopic expression of DACT1α in BGC803, MGC823 and AGS cell lines was confirmed by Western blot (cropped gel). (**B2**) Knockdown effect of DACT1α by shDACT1α in normal gastric epithelial cell line GES1 was confirmed by Western blot (cropped gel). (**C**) pcDNA3.1-DACT1α vector stably transfected BGC823 and MGC803 cells were significantly slowed the spreading rate on cover slips for 6 hours compared to control vector pcDNA3.1 transfected and BGC823 and MGC803 cells. (**D**) BGC823 and MGC803 cells stained with rhodamine-phalloidin for detecting F-actin formation.

DACT1α was expressed in normal human gastric epithelial cell GES1 and in normal stomach tissue, but down-regulated or silenced in gastric cancer cell lines both at mRNA and protein level (Figure 1A).

### DACT1α inhibits of gastric cancer cell spreading

The frequent silence of DACT1α in gastric cancer cell lines but not in normal gastric mucosa suggests that DACT1α may act as a tumor suppressor. In this connection, we examined the effect of ectopic expression of DACT1α in gastric cancer cell lines. Three gastric cancer cell lines (AGS, BGC823 and MGC803) with different differentiation and characteristics were selected for functional evaluation of DACT1 *in vitro*. *AGS* is a well-differentiated gastric epithelial *cell line; BGC823* is an undifferentiated gastric epithelial cell line and *MGC803 is* a poorly differentiated mucoid stomach adenocarcinoma cell line. Re-expression of DACD1 in the stably transfected BGC823, MGC803 and AGS cells was confirmed by western blot (Figure 1B1). Ectopic expressing DACT1α in silenced gastric cancer cell lines BGC823 and MGC803 cell lines significantly inhibited in cell spreading in comparison with control cells by stably transfectants with DACT1α in BGC823 and MGC803 cell lines (BGC823, *P* = 0.003; MGC803, *P* = 0.001) (Figure 1C). DACT1α also down-regulated actin microfilament (stress fiber) formation as determined by staining with rhodamine-labeled phalloidin (Figure 1D).

### DACT1α suppresses gastric cancer cell migration

**Figure 2:**
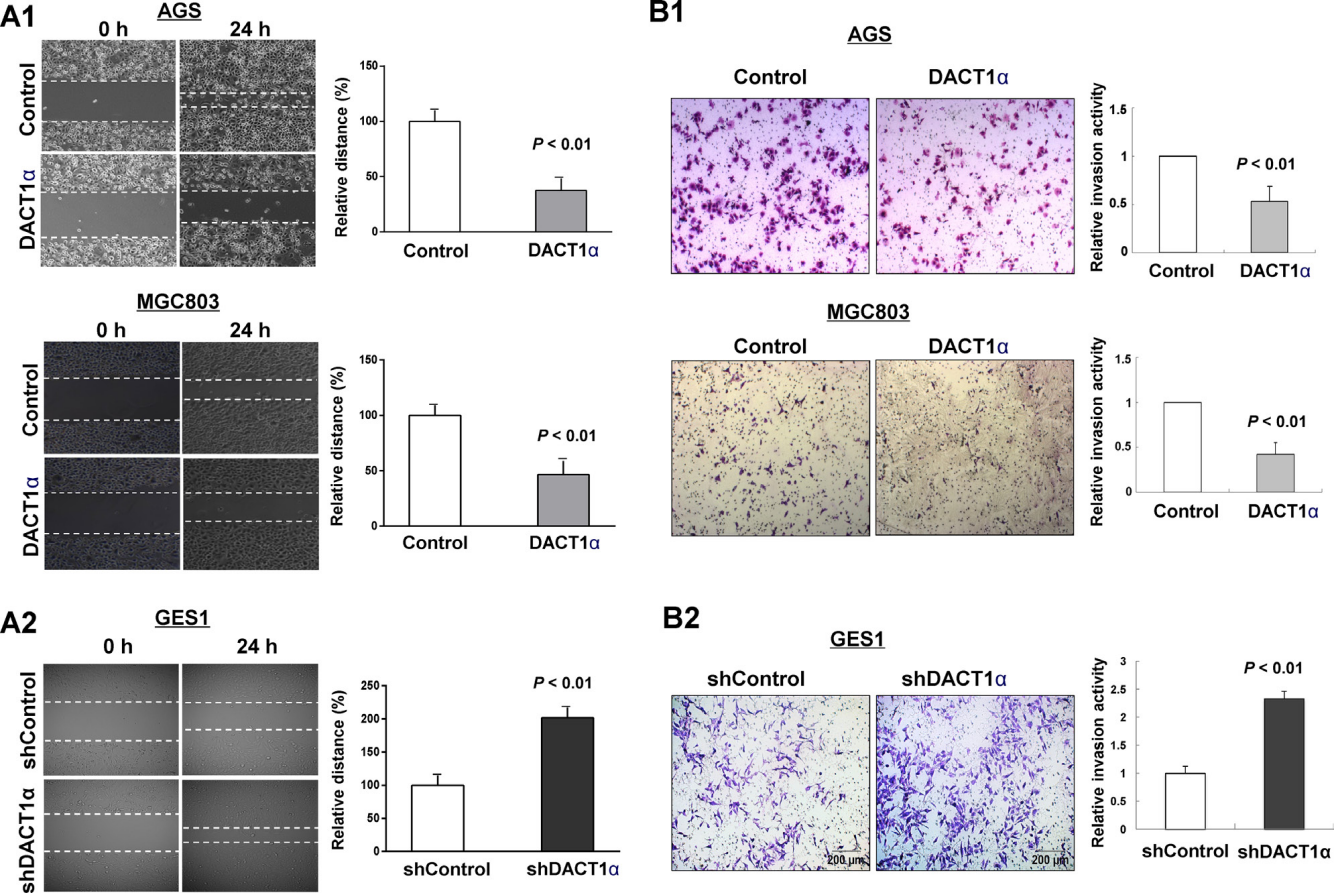
DACT1α inhibited gastric cancer cell migration and invasion ability (**A1**) Cell migration rates of pcDNA3.1 and pcDNA3.1-DACT1α vector transfected AGS and MGC803 cells were compared via wound healing assays. (**A2**) Knockdown of DACT1α by shDACT1α promoted wound healing closure rate. Microscopic observation was recorded at 0 and 24 hours after scratching the surface of a confluent layer of cells. (**B1**) Invasion rates of pcDNA3.1 and pcDNA3.1-DACT1α vector transfected AGS and MGC803 cells. (**B2**) Knockdown of DACT1α by shDACT1α promoted GES1 cell invasion. Number of cells that invaded through the Matrigel was counted in 10 fields under the ×20 objective lens.

*In vitro* wound healing assay showed that ectopic expression of DACT1α markedly slowed cell migration at the edges of scratch wound of AGS and MGC803 (Figure 2A1), while an inverse effect was observed in GES1 with knockdown of DACT1α (Figure 2A2). Quantitative analyses at 24 h confirmed a significant reduction in wound closure in DACT1α-transfected AGS cells (*P* < 0.01) and MGC803 (*P* < 0.05) compared with empty vector-transfected control cells (Figure 2A1). On the other hand knockdown DACT1α in normal gastric epithelial cell line GES1 was confirmed by Western blot (Figure 1B2). Knockdown DACT1α in GES1 cells significantly promoted wound closure rate compared with the control cells (*P* < 0.01) (Figure 2A2).

### DACT1α suppresses gastric cancer cell invasion

We further evaluated the effect of DACT1α in cell invasion. We found that ectopic expression of DACT1α significantly dampened cell invasion ability as measured by transwell assays in AGS ((*P* < 0.01) and MGC803 (*P* < 0.01) cells stably transfected pcDNA3.1-DACT1α compared to stably transfected control vector, respectively (Figure 2B1). Inversely, knockdown DACT1α induced GES1 cell invasive ability (Figure 2B2).

### DACT1α regulates planar cell polarity (PCP) pathway

**Figure 3:**
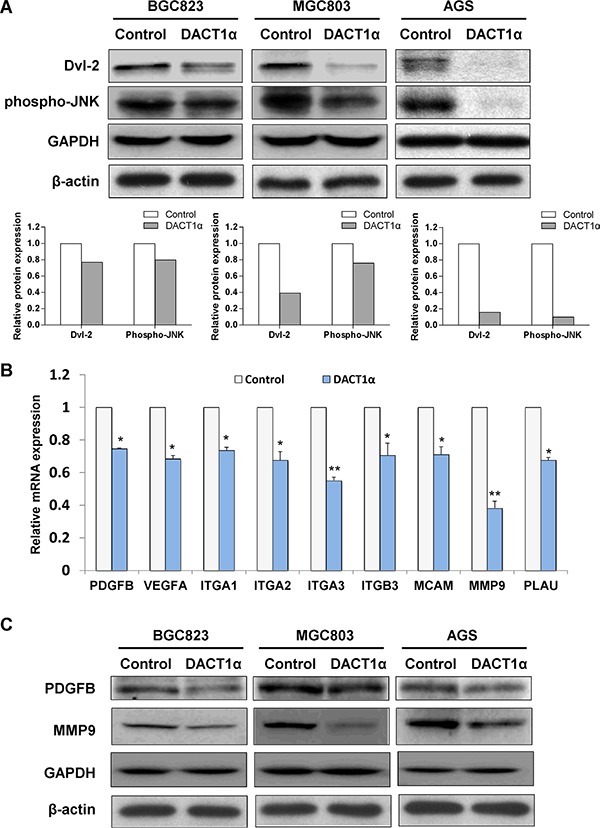
(**A**) DACT1α regulated PCP pathway by promoting Dvl-2 degradation and suppressing the activation of JNK pathway in AGS cells (cropped gel). (**B**) Real-time PCR validation for gene expression profile in DACT1α stably transfected BGC823 cells screened by Cancer Pathway cDNA microarray. (**C**) Two represented genes PDGFB and MMP9 expression decreased in DACT1α over-expressed GC cell lines (cropped gel).

To examine whether DACT1α regulated PCP pathway in gastric cancer cells [11, 12, 15], expression of central PCP component Dvl-2 and activation of PCP downstream JNK pathway were determined by western blot in pcDNA3.1 and pcDNA3.1-DACT1α stably transfected BGC823, MGC803 and AGS cells. As shown in Figure 3A, DACT1α regulated PCP pathway by promoting Dvl-2 degradation and suppressing the active form of JNK in GC cells.

### Downstream target cancer-related genes regulated by DACT1α

**Figure 4:**
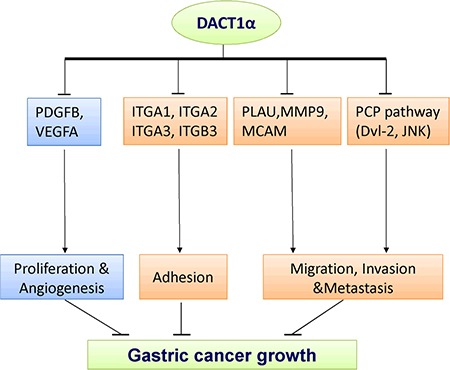
The molecular mechanisms underlying the tumor suppression of DACT1α in gastric cancer Schematic diagram for the molecular basis of DACT1α as a tumor suppressor gene in gastric cancer. Ectopic expression of DACT1α suppressed gastric cancer cell growth was associated with several biological effects: 1) Suppression of PDGFB and VEGFA contributed to dampened cell proliferation and angiogenesis. 3) DACT1α mediated cell spreading adhesion, migration and invasion inhibition were associated with the suppression of ITGA1, ITGA2, ITGA3, ITGB3, MCAM, MMP9 and PLAU.

To gain insight into the molecular mechanisms underlying the tumor suppression of DACT1α, gene expression profile in DACT1α stably transfected BGC823 were analyzed by Cancer Pathway cDNA microarray and further validated by real-time PCR (Figure 3B). Two representative genes PDGFB and MMP9 which included in proliferation and migration pathways were further verified by western blot (Figure 3C). Compared to control vector transfected cells, the anti-tumorigenesis effect by DACT1α was mediated by regulating important genes in cell proliferation, angiogenesis, adhesion, migration and invasion (Table 1). DACT1α led to down-regulation of angiogenic platelet-derived growth factor beta polypeptide (PDGFB), vascular endothelial growth factor A (VEGFA) as well as multiple cell migration and invasion molecules integrin, alpha 1 (ITGA1), integrin, alpha 2 (ITGA2), integrin, alpha 3 (ITGA3), integrin, beta 3 (ITGB3), melanoma cell adhesion molecule (MCAM), matrix metallopeptidase 9 (MMP9) and plasminogen activator, urokinase (PLAU) (Figure 4).

**Table 1:** The effect of DACT1α on gene expression profiles of cancer pathways

Genebank Accession	Gene Symbol	Gene Name	Gene Function	Fold Change (DACT1/control)
NM_002608	PDGFB	Platelet-derived growth factor beta polypeptide	Proliferation &Angiogenesis	−1.5
NM_003376	VEGFA	Vascular endothelial growth factor A	Proliferation &Angiogenesis	−1.5
NM_181501	ITGA1	Integrin, alpha 1	Adhesion	−1.5
NM_002203	ITGA2	Integrin, alpha 2	Adhesion	−1.5
NM_002204	ITGA3	Integrin, alpha 3	Adhesion	−1.5
NM_000212	ITGB3	Integrin, beta 3	Adhesion	−1.6
NM_004994	MMP9	Matrix metallopeptidase 9	Invasion & Metastasis	−1.5
NM_006500	MCAM	Melanoma cell adhesion molecule	Invasion & Metastasis	−1.5
NM_002658	PLAU	Plasminogen activator, urokinase	Invasion & Metastasis	−1.8

### Association of DACT1α methylation with the survival of gastric cancer patients

**Figure 5:**
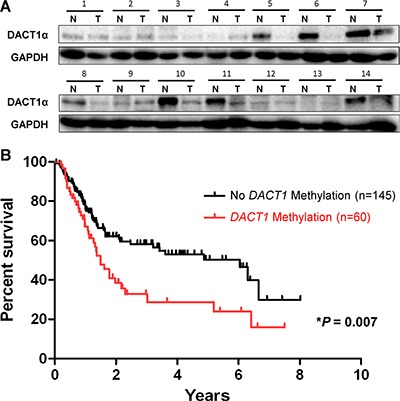
Kaplan-Meier survival curves show that gastric cancer patients with DACT1α methylation had poorer survival than those without DACT1α methylation (**A**) Expression of DACT1α protein was significantly down-regulated in GC tumor tissues compared to adjacent non-tumor tissues (*n* = 14) (normalized to GAPDH) (cropped gel). (**B**) Patients with DACT1α hypermethylation had poor survival rate than those without methylation (U) based on the log-rank test (*P* = 0.007).

DACT1α protein expression level was reduced in gastric cancer tissues compared with their matched adjacent normal tissues by Western blot (Figure 5A). DACT1α methylation was evaluated in 20 normal gastric biopsies and 205 primary gastric cancer tissues by direct bisulfite genomic sequencing. None was detected to have DACT1α methylation in 20 healthy gastric tissue samples. DACT1 methylation was associated with advanced tumor size grade (*P* < 0.05), lymph node metastasis (*P* < 0.05) and distant metastasis (*P* = 0.05) respectively, but was not associated with the clinicopathological characteristics of patients with gastric cancer including age, gender, *H. pylori* infection, Lauren classification and tumor differentiation. Among 205 gastric cancer cases, we found that promoter methylation of DACT1α was detected in 29.3% (60/205) of gastric cancers, but not in normal controls. Overall survival of the gastric cancer patients was analyzed for dependence on DACT1α promoter methylation using Kaplan-Meier survival curves. The overall survival of patients with DACT1α methylation was significantly shorter than that of other gastric cancer patients (*P* = 0.007, Figure 5B). Using univariate Cox regression analysis, DACT1α methylation was associated with a significantly increased risk of cancer-related death (*P* = 0.007, Table 2). In the multivariate Cox regression analysis, as expected, TNM stages were significantly associated with the survival of gastric cancer patients. DACT1α methylation was associated with the poor outcome in gastric cancer patients (Table 3).

**Table 2:** Univariate Cox regression analysis of potential prognostic factors for gastric cancer patients

Variable	HR (95% CI)	*P* value
**Age**	1.01 (0.99–1.03)	0.50
**Gender**		
Male	1.07 (0.70–1.64)	0.77
Female	1.00	
***H. pylori infection***		
Negative	1.46 (0.80–2.68)	0.22
Positive	1.00	
**Lauren**		
Intestinal	1.25 (0.66–2.52)	0.53
Diffuse	1.83 (0.72–4.66)	0.20
Mixed	1.00	
**TNM stage**		
I	0.08 (0.02–0.24)	< 0.0005
II	0.14 (0.06–0.32)	< 0.0005
III	0.27 (0.16–0.44)	< 0.0005
IV	1.00	
***DACT1 methylation***		
Yes	1.86 (1.08–2.82)	0.007
No	1.00	

**Table 3:** Multivariate Cox regression analysis of potential prognostic factors for gastric cancer patients

Variable	HR (95% CI)	*P* value
**Age**	1.01 (0.99–1.03)	0.33
**Gender**		
Male	1.39 (0.87–2.24)	0.073
Female	1.00	
**TNM stage**		
I	0.07 (0.02–0.21)	< 0.0005
II	0.12 (0.05–0.29)	< 0.0005
III	0.24 (0.14–0.40)	< 0.0005
IV	1.00	
***DACT1 methylation***		
Yes	1.712 (1.034–2.87)	0.047
No	1.00	

## DISCUSSION

In this study, we found that the mRNA expression of DACT1α was silenced or reduced in 100% (8 of 8) gastric cancer cell lines, but expressed in normal gastric cell line and tissue. These results suggested that DACT1α may play a tumor suppressive role in gastric cancer.

As the functional role of DACT1α in cell migration and invasive abilities has not been well evaluated, we examine the effect of DACT1α in mediating the cell migration and invasive abilities in gastric cancer cell lines. For metastasis functional study, we found that DACT1α led to a significant decrease of cell spreading ability in BGC823 and MGC803 cells. At the beginning of cell spreading, most of DACT1α-re-expressed cells maintained a spherical shape whereas a large population of control cells had flatten out and spread onto the matrix. Cell spreading is the first step for cancer cell migrating and invading neighboring tissue. In order for cells to move and invade, they must adhere, flatten out and spread onto the extracellular matrix with extended protrusions. In keeping with reduced cell spreading ability, DACT1α significantly dampened cell migration and invasion as demonstrated by wound healing assay and trans-well invasion assay.

DACT1α was an important regulator in PCP pathway [11, 12, 16]. Increasing evidences have demonstrated that PCP pathway played important roles in cancer development especially in tumor metastasis and angiogenesis [17]. Dishevelled (Dvl) 2, a core PCP component was over-expressed in non-small cell lung cancer and colorectal cancer and correlated to poor tumor differentiation and advanced TNM stages [18, 19]. Genetic deletion of Dvl2 reduced the intestinal tumor numbers in a dose-dependent way in the Apc(Min) model for colorectal cancer [19]. Therefore DACT1α could inhibit tumor metastasis partially depending on PCP pathway.

In order to elucidate the other downstream targets modulated by DACT1α in tumor inhibition, cancer pathway cDNA microarray was performed in BGC823-DACT1α cell line. 9 markers covering cell proliferation, adhesion, migration, invasion and angiogenesis were involved in the anti-tumorigenesis effect of DACT1α in gastric cancer. PDGFB and VEGFA were angiogenic factors and mediated tumor growth, angiogenesis and metastasis [20–23]. Therefore, down-regulation of PDGFB and VEGFA may explain the anti-proliferative effect exerted by DACT1α in gastric cancer cells.

The reduced cell motility and spreading effect caused by DACT1α in gastric cancer cells was revealed to be at least associated with altered PCP signaling pathway and JNK activity. DACT1α promoted central PCP component Dvl-2 degradation and suppressed PCP downstream pathway JNK activity in AGS cells. Consistently, DACT1α knockout mice showed disregulated PCP signaling pathways by post-translationally antaganizing Dvl2 and Vangl2 [11, 12, 15]. JNK pathway and RhoA pathway are two main PCP pathways affected by DACT1α, both of which controlled cytoskeleton reorganization in cell movements and polarity [11, 12]. Less active RhoA but more active JNK was found in mouse embryonic fibroblast derived from DACT1α knockout mice [11, 12], Notably, it has been well accepted that RhoA normally allowed the cell to maintain a spherical shape in the process of spreading whereas RhoA-depleted cells showed enhanced elongated and extended protrusions, permitting cells to flatten out, spread and invade much faster [24–26]. Therefore, DACT1α over-expression modulated PCP pathways, contributing to decreased cell spreading activity, F-actin assembly, cell migration and invasion abilities.

Cancer Pathway cDNA microarray analysis revealed that DACT1α down-regulated cell adhesion receptors from the integrin family including ITGA1, ITGA2, ITGA3 and ITGB3. Integrins spanned the cell membrane and controlled adhesion to the extracellular matrix. Each integrin consisted of two subunits: α and β. One of the most well-studied integrins in cancer was oncogenic integrin α_v_β_3_, of which ITGB3 (also known as β3 integrin) was a key component [27]. Integrin α_v_β_3_ was usually expressed at low or undetectable levels in most adult epithelia, but can be highly upregulated on the most aggressive tumor cells in a variety of cancer [27]. The expression of ITGB3 was restricted exclusively to cells within vertical growth phase and metastatic melanomas and was correlated with shorter survival [28]. Several integrin α_v_β_3_ antagonists have been proved to be efficient in reducing tumor growth and metastasis in many mouse models and clinical studies [27]. In a mouse model of spontaneous tumorigenesis caused by WNT1 overexpression, the luminal epithelial progenitor marker ITGB3 identified a highly tumorigenic cancer stem cell population [29]. By recruiting c-Src to ITGB3 cytoplasmic tail, integrin α_v_β_3_ activated c-Src, substantially increased anchorage-independent tumor cell survival *in vitro* and metastasis *in vivo* [27]. In addition, DACT1α decreased the mRNA expression of ITGA1 and ITGA2, which were components of collagen receptor integrin α_1_β_1_ and α_2_β_1_. Integrin α_1_β_1_ and α_2_β_1_ were found to be important regulators in cell invasion in human cancers [30, 31]. Taken together, those down-regulated integrin molecules by DACT1α may at least offer partial explanation for impaired cell adhesion spreading ability, migration and invasion.

Other cell migration and invasion molecules regulated by DACT1α included PLAU, MCAM and MMP9. PLAU played important functions in cell migration, invasion and survival [32]. Elevated PLAU expression was found in many human cancers and correlated with the clinical features of biologically aggressive cancer and poor outcome of cancer patients [33, 34]. By binding urokinase-type plasminogen activator receptor (uPAR), PLAU promoted extracellular matrix (ECM) proteolysis and generated the protease plasmin which cleaved a range of ECM components and activated growth factors and matrix metalloproteases such as MMP9 [35]. Moreover, activated uPAR signals interacted with ITBG3 and triggered intracellular Rac signaling pathway for cell migration, leading to increased F-actin assembly and membrane protrusion [35]. Besides, MMP9 and MCAM were well-established metastasis mediators which actively involved in pathological processes, such as metastasis [36, 37].

To assess the clinical application of DACT1α in gastric cancer, we examined the promoter methylation of DACT1α by direct bisulfite sequencing in 205 primary gastric cancer patients and 20 normal controls. We found that promoter methylation of DACT1α was detected in 29.3% (60/205) of gastric cancers, but not in normal controls. This result indicated that methylation mediated silencing of DACT1α is a frequent event in gastric cancer. Moreover, in keeping with the dampened migration and invasive ability of gastric cancer cells by DACT1α *in vitro*, DACT1α methylation was significantly correlated with patient survival by Kaplan–Meier curves. Moreover, multivariate Cox regression analysis revealed that after adjustment for TNM stage, age and gender, gastric cancer patients with DACT1α methylation showed poorer survival in gastric cancer patients (*P* < 0.05), suggesting that DACT1α methylation may serve as an independent prognostic factor of gastric cancer patient.

In conclusion, we have identified a functional tumor suppressor gene DACT1α inhibiting cell migration and invasion in gastric cancer. The impaired cell adhesion spreading ability, migration and invasion by DACT1α are mediated through down-regulated integrin molecules. DACT1α methylation may serve as a potential epigenetic biomarker to predict outcome of gastric cancer patients.

## MATERIALS AND METHODS

### Cell lines

Gastric cancer cell lines (AGS, Kato III, MKN28, MKN45, NCI87, SNU1, BGC823 and MGC803) and normal gastric epithelial GES1 cell line were obtained from American Type Culture Collection (Manassas, VA) and Beijing Oncology Hospital (China). Cell lines were maintained in RPMI-1640 or Dulbecco's modified Eagle's medium (DMEM) medium (Gibco BRL, Rockville, MD) with 10% fetal bovine serum. Human normal adult tissue RNA samples were purchased commercially (Stratagene, La Jolla, CA or Millipore Chemicon, Billerica, MA).

### Human tissue samples

Gastric tissues samples were obtained from 205 Chinese gastric cancer patients and 20 normal controls. All patients diagnosed to have gastric cancer received surgical resection of the tumor. Clinical data of patients were collected from medical record and structured interview of patients. Clinical information included age, gender, survival time, TNM stage (I-IV), tumor size (I-IV), lymph node status (postive or negative), distant metastasis (postive or negative), differentiation (poor, moderately or well differentiated), *H. pylori* infection, tumor location, Lauren classification and chemotherapy. The informed consent was obtained from the patients and the study protocol was approved by the Clinical Research Ethics Committee of the First Hospital, Hebei Medical University and the methods were carried out in “accordance” with the approved guidelines.

### Bisulfite modification of DNA and bisulfite genomic sequencing

Genomic DNA was extracted from fresh gastric tissues of healthy subjects or paraffin-embedded tissues of gastric cancer patients using DNA mini kit (Qiagen, Hilden, Germany). Extracted DNA was treated with sodium bisulfite using an EZ DNA Methylation-Gold^™^ Kit (Zymo Research, Orange, CA). The bisulfite-modified DNA was amplified by using primer pairs that specifically amplify either methylated or unmethylated sequences of the DACT1α gene. The PCR product was separated by electrophoresis on 1% agarose with ethidium bromide staining. The amplicon were purified by using the ExoSAP-IT PCR Clean-up Kit for the removal of unwanted primers and dNTPs (GE Healthcare, Pittsburgh, PA). PCR product was mixed with ExoSAP-IT at the ratio of 5:2 and incubated for 15 min at 37°C followed by enzyme inactivation at 80°C for a further 15 min. The purified DNA was examined by direct sequencing PCR with the Big Dye Terminator Cycle Sequencing kit (Applied Biosystems). Sequencing PCR product was detected by ABI PRISM 7000 Sequence Detection System (Applied Biosystems).

### RNA extraction, semi-quantitative RT-PCR and real-time PCR analyses

Total RNA was extracted by TRIzol Reagent (Molecular Research Center Inc, Cincinnati, OH) from cell pellets or tissues. cDNA was synthesised by Transcriptor Reverse Transcriptase (Roche, Indianapolis, IN). Semi-quantitative RT-PCR was performed with AmpliTaq Gold DNA polymerase (Applied Biosystems, Foster City, CA). β-actin was employed as internal control. Real-time PCR was performed with SYBR Green master mixture (Roche, Indianapolis, IN) on LightCycler^®^ 480 Instrument. 10ng cDNA template and 3pmol of primer pairs were applied in each 15μl PCR reaction. Each sample was tested in triplicate.

### Construction of DACT1α expression plasmid

The full-length open reading frame sequence of DACT1α was obtained by RT-PCR amplification of normal human gastric cDNA. DACT1 was cloned in pcDNA3.1+ and pBABE using primers as below:

**Table:** 

c-FLAG-DACT1a-F2	GGactagtATGgactataag gacgatgatgacaagAAGCC GAGTCCGGCC
c-DACT1alpha-R2	ccgctcgagTCAAACCGT CGTCATCAGTTTC
pBABE-DACT1-F	AAAGGCAtacgtaATGA AGCCGAGTCCGGC
pBABE-DACT1-R	ACGCgtcgacTCAAACCG TCGTCATCAGTTTC

The PCR aliquots were subcloned into the mammalian expression vector pcDNA3.1 and then verified by DNA sequencing.

### Establishment of stable DACT1α-expressing cells

BGC823, MGC803 or AGS cells were transfected with pcDNA3.1 or pcDNA3.1-DACT1α plasmid using lipofectamine 2000 (Life Technologies). Stable transfections were selected for 2 weeks with G418 antibiotics. For retroviruses production, 293FT cells (Invitrogen) were co-transfected with pBABE-puro-DACT1 or pBABE-puro empty vector, two packaging plasmids pUMVC (Addgene) and pCMV-VSV-G (Addgene) at the ratio of 1: 0.9: 0.1. At 24 hours post-transfection, cells were feeded with fresh medium. After 48 hour post-transfection, the supernatant containing retrovirus pBABE-puro-DACT1α or pBABE-puro control was harvested and stored at −80°C. To generate stable DACT1α expressing or control cell line, retrovirus pBABE-puro-DACT1α or pBABE-puro control-containing supernatant was added to cells. After 48 hour transduction, the media containing retrovirus was removed and replaced with fresh medium containing 0.5 ug/ml antibiotic puromycin (Invitrogen) for selection.

### RNA interference and transfection

Knock-down DACT1α expression in GES1 cell lines was performed by a shRNA targeting DACT1α. Both GES1-shRNA control and GES1-DACT1α shRNA cells were selected for 2 weeks with G418 after transfection 48 h. The cells were ready for further experiments.

### Cell spreading assay

BGC823 and MGC803 cells stably transfected with pcDNA3.1-DACT1α or pcDNA3.1 were collected by trypsinization, washed twice with DMEM medium and were resuspended on cover slips and culture plate. Cells were allowed to spread for 6 hours in DMEM containing 10% FBS at 37°C and then photographed. The cell spreading rate was calculated as: number of spreading cells / number of total cells (%). The cover slips with spreading cells were fixed with 3% paraformaldehyde for F-actin staining. The experiments were performed in triplicate at three independent experiments. Every experiment was performed by using the different batches of transfection.

### F-actin staining

Cell spreading assay was performed using BGC823 and MGC803 cells stably transfected with pcDNA3.1-DACT1α or pcDNA3.1. The cover slips with spreading cells were fixed with 3% paraformaldehyde (in PBS) at room temperature for 15 min. Then the cells were washed three times with 1 × TBS and permeabilized with 0.1% Triton X-100 at room temperature for 5 min. Then cells were washed three times with 1 × TBS and blocked with 1% BSA at room temperature for 30 min. For F-actin staining, cells were stained with Rhodamine phalloidin (1:40 dilution in 0.2% BSA) (Invitrogen) at 37°C for 20 min in dark. Finally, were washed and mounted with Mounting Medium containing DAPI (Vector Laboratories). The experiments were performed in triplicate at three independent experiments.

### Wound-healing assay

To carry out the wound healing assay, AGS and MGC803 stably transfected with pcDNA3.1-DACT1α or pcDNA3.1 control vector were seeded into 6-well culture plates. After 24 h, the monolayer AGS and MGC803 cells were then scratched manually with a plastic pipette tip, and after being washed with PBS, the wounded monolayers of the cells were allowed to heal for 24 hours in reduced serum RPMI 1640 and DMEM medium. Photographs were taken at 0 and 24 h respectively. The experiments were performed in triplicate at three independent experiments.

### Invasion assay

To measure the cell invasion activity, Transwell assays were done using a BD BioCoat^™^ Growth Factor Reduced MATRIGEL^™^ Invasion Chamber (Cat. No. 354483, BD Biosciences). AGS and MGC803 cells were seeded into six-well dishes. After 24 h, the cells were transfected with pcDNA3.1-DACT1α or the empty control vector. After 48 h transfection, AGS and MGC803 cells (2.5 × 10^4^ in 500 uL) suspended in serum-free RPMI 1640/DMEM containing 0.1% bovine serum albumin were applied to the upper chamber. RPMI 1640/DMEM containing 20% fetal bovine serum was added to the lower chamber. After the cells were incubated at 37°C for 24 hours, the number of cells that migrated to the lower side of the upper chamber was counted. Three independent experiments were performed and results were shown as the means ± s.d. The experiments were performed in triplicate at three independent experiments.

### Cancer pathway finder PCR array and real-time PCR validation

Gene expression profiles of BGC823 cells stably transfected with pcDNA3.1- DACT1α or pcDNA3.1 empty vector were analyzed by a commercial gene expression array system named the Human Cancer PathwayFinder^™^ RT^²^ Profiler^™^ PCR Array (96-well) (SABiosciences, Frederick, MD). This array contains 84 functionally well characterized genes representative of the six biological pathways involved in human tumorigenesis (Supplementary Table S1). For real-time PCR validation analysis, Ct was measured during the exponential amplification phase, and the amplification plots were analyzed using SDS 1.9.1 software (Applied Biosystems). The relative expression level (defined as fold change) of target gene is given by 2^–ΔΔCt^ (ΔCt = ΔCt^target^ – ΔCt^βactin^; ΔΔCt = ΔCt^DACT1α-expressing^ – ΔCt^control^) and normalized to the fold change detected in the corresponding control cells, which was defined as 1.0. All reactions were performed in duplicate. Primer sequences of target genes are listed in Supplementary Table S2.

### Statistical analysis

The results were expressed as mean ± standard deviation (SD). The difference in *in vitro* functional studies between the two groups was determined by the Student *t* test. Kaplan-Meier survival curves for trend were used to evaluate the relationship between DACT1α methylation and the prognosis from the date of primary diagnosis to the end of follow-up. The analysis was performed using SPSS statistical package for Windows (version 16; SPSS). A *P* value of less than 0.05 was taken as statistical significance.

## SUPPLEMENTARY MATERIALS


