# Supplementary material for: Dapper homolog 1 alpha suppresses metastasis ability of gastric cancer through inhibiting planar cell polarity pathway
Source: Oncotarget. 2016 Nov 9;7(49):81423–34. doi: 10.18632/oncotarget.13234 (PMC5348403; doi:10.18632/oncotarget.13234)
Supplement: Supplementary file 1 [file oncotarget-07-81423-s001.pdf]

Dapper homolog 1 alpha suppresses metastasis ability of gastric cancer through inhibiting planar cell polarity pathway

Supplementary Materials

Supplementary Table S1: List of cancer-related genes representative of the six biological pathways in human cancer pathway finder array

Cell Cycle Control & DNA Damage Repair:

ATM, BRCA1, CCNE1 (cyclin E1), CDC25A, CDK2, CDK4, CDKN1A (p21Waf1), CDKN2A (p16Ink4), CHEK2 (chk2 / Rad53), E2F1, MDM2, RB1, S100A4, TP53 (p53)

Apoptosis and Cell Senescence:

APAF1, BAD, BAX, BCL2, BCL2L1 (bcl-X), CASP8, CFLAR (CASPER), GZMA, HTATIP2, TERT (telomerase), TNFRSF1A (TNF-a receptor), TNFRSF10B (DR5), TNFRSF25 (DR3)

Signal Transduction Molecules and Transcription Factors:

AKT1, ERBB2, ETS2, FOS, JUN, MAP2K1 (MEK), MYC, NFKB1 (NFκB), NFKBIA (IκBα), PIK3R1 (PI3K p85α), RAF1, SNCG

Adhesion:

ITGA1 (integrin α1), ITGA2 (integrin α2), ITGA3 (integrin α3), ITGA4 (integrin α4), ITGAV (integrin αV), ITGB1 (integrin β1), ITGB3 (integrin β3), ITGB5 (integrin β5), MCAM, MTSS1, PNN, SYK, UCC1

Angiogenesis:

ANGPT1 (angiopoietin-1), ANGPT2 (angiopoietin-2), COL18A1 (endostatin), FGFR2, IFNA1 (IFNα), IFNB1 (IFNβ), IGF1, IL8, PDGFA, PDGFB, TEK (tie-2), TGFB1, TGFBR1 (ALK-5), THBS1 (thrombospondin-1), TNF, VEGFA

Invasion and Metastasis:

MET, MMP1 (collagenase-1), MMP2 (gelatinase A), MMP9 (gelatinase B), MTA1, MTA2, NME1, NME4, PLAU, PLAUR, S100A4, SERPINB5 (maspin), SERPINE1 (PAI1), TIMP1, TIMP3, TWIST1

Supplementary Table S2: List of real-time PCR primers for validation

Primer Name	Sequence (5′-3′)	Annealing Temperature
β-actin-F	GTCTTCCCCTCCATCGTG	60
β-actin-R	AGGGTGAGGATGCCTCTCTT	
ITGA1-F	GTCAGCCCCACATTTCAAGT	60
ITGA1-R	GGAACCATCCAGCACTATGAC	
ITGA2-F	TTGACCTATCCACTGCCACA	60
ITGA2-R	CTCCAGTTCCCATGTTCCTG	
ITGA3-F	TCCATCGGCAGACAGAGC	60
ITGA3-R	GCACAGGTACACAGCACCAG	
ITGB3-F	CTCAAGTCAGTCCCCAGAGG	60
ITGB3-R	TCCACAGGGTAATCCTCCAC	
VEGFA-F	CTACCTCCACCATGCCAAGTG	60
VEGFA-R	TGATTCTGCCCTCCTCCTTCT	
PDGFB-F	ATCCGCTCCTTTGATGATCT	60
PDGFB-R	GGGTCATGTTCAGGTCCAAC	
IL8-F	GAACTGAGAGTGATTGAGAGTGGA	60
IL8-R	CTCTTCAAAAACTTCTCCACAACC	
MMP9-F	CCTGGAGACCTGAGAACCAATC	58
MMP9-R	CCACCCGAGTGTAACCATAGC	
PLAU-F	AACGTACCATGCCCACAGAT	60
PLAU-R	TCTTGGACAAGCGGCTTTAG	
